# Cognitive Intervention Programs in Minors Belonging to Disadvantaged Contexts in Spain: A Systematic Review

**DOI:** 10.3390/children9091306

**Published:** 2022-08-28

**Authors:** Carmen García-Navarro, Rosalba Company-Córdoba, Antonio Sianes, Joaquín A. Ibáñez-Alfonso

**Affiliations:** 1 Research Institute on Policies for Social Transformation, Universidad Loyola Andalucía, 14004 Córdoba, Spain; 2 Human Neuroscience Lab, Department of Psychology, Universidad Loyola Andalucía, 41704 Sevilla, Spain

**Keywords:** low socioeconomic status, poverty, 2030 agenda, children, adolescents, cognitive training, neuropsychological intervention, cognitive development

## Abstract

Research studies show a strong influence of socioeconomic status (SES) on human development, and how the exposure to risk contexts in the earliest stages translates into dangers in the cognitive development of children and adolescents. To alleviate these consequences and favour development, different cognitive training programs have contributed to this field by identifying the criteria of efficacy. This systematic review identifies and synthesizes the evidence of cognitive intervention studies implemented with psychosocial risk groups carried out in Spain. The search strategy was adapted to different databases. Only studies published in English or Spanish and developed in Spain that included interventions applied in populations aged 5 to 18 years with a low SES were included. The analysis of the literature showed nine interventions that indicated an improvement in those cognitive functions worked with low SES children. The cognitive domains that most worked were executive functions, followed by social cognition and language. After reviewing the available literature, a clear scarcity of interventions carried out in Spain was observed. Variables such as age, cognitive functions or personal vulnerability were identified as factors to be taken into account in future lines of research due to their influence on minors. These findings indicate the relevance of this review to help decision-making in relation to the actions to be carried out by the competent bodies in Spain.

## 1. Introduction

Today, 10% of the world’s population lives in poverty, and access to basic needs such as education or health care is difficult. With the objective of responding to these needs, the United Nations (UN) has launched an action plan, proposing a set of sustainable development goals (SDGs) for nations to meet by 2030 [1]. These objectives include the eradication of poverty as a cause of other problems.

It is of vital importance to consider the variables that can influence children’s development and to investigate the effectiveness of various interventions. Since the outbreak of COVID-19, these goals have become even more urgent. The World Bank [2] estimated that approximately 365 million children, or 1 in 6 minors, lived in extreme poverty before the pandemic. With its worldwide economic consequences, the pandemic is expected to increase this number. In Spain, the general risk of poverty is 20.7%, and in the population under 16 years of age, it is 27.1%, according to the Survey of Living Conditions of 2019 [3].

The concept of poverty from a classical point of view is linked to economic inequality and refers only to the low-income population. However, in recent years, studies have indicated that this population presents mechanisms of marginalization in which other factors, in addition to economics, lead them to experience social exclusion [4].

Since the United Nations Development Program (UNDP) of 2016 [5], this new concept of poverty in which other dimensions are taken into account, has been named multidimensional poverty. Multidimensional poverty includes two main concepts: economic well-being, since the victims of this poverty do not have sufficiently high incomes to cover their needs, and social rights, since due to socioeconomic characteristics this population is vulnerable to deficiencies in food, education, housing or health [6]. In addition, the impact of poverty on the population is arduous, since it also implies exposure to risk factors resulting from environmental deficiencies [7]. Therefore, it is important to consider individual, environmental and cultural factors that affect this population [7]. Thus, the sphere of culture is considered from a transversal point of view, since it is a predictor of the social structure and, therefore, of the type of laws and economy of a place. These aspects influence the reality of families and determine their economic well-being, profession, health and education [8], aspects that contribute to the socioeconomic status (SES) of a family [9].

This theoretical construct is closely related to the adequate development of children, since family SES is a predictor of a child’s physical, emotional and cognitive development. SES also influences the conditions to which minors are exposed. Bäckman and Nilsson [10] mention that the risk factors most commonly related to low income are violence, lack of stimulation, stress, lack of support, social exclusion and substance abuse [8].

In the case of minors, exposure to these factors affects development. This is because human development is governed not only by genetics but also by factors that stimulate it and are present in the family and social environment [11]. Thus, development is made possible by genes, limited by sensitive periods or maturation, and determined by environmental stimulation in early childhood [12]. Its evolution is multidimensional and multidirectional. Development of domains, such as cognitive, socioemotional and physical, will occur at a different life stage depending on the characteristics of individuals and their current environment and the one in which they grew up [11,12].

Cognitive domains are mental processes that allow us to carry out the activities of daily life. These domains help to receive, process and execute actions based on the information an individual has in that moment. Some of the cognitive domains are attention, memory, language, social cognition and executive functions [13,14]. Different studies affirm that cognitive development is dependent on family SES, since there is an affectation in the prefrontal lobe of the brain due to exposure to stress in the first years of life. General cognitive functions, such as language or emotional processes are located here, with executive functions such as working memory, cognitive flexibility and inhibition [15,16].

The areas most affected are usually those related to executive functions and language, as they are interrelated and have a longer maturation period [15]. It is considered that executive functions continue to mature during adolescence, and there have even been improvements in performance in complex tasks of inhibition and flexibility, which is in line with what we know about the physiological processes of maturation of the prefrontal cortex. This is mainly linked to executive functions, in which maturation is completed at approximately 25 years [11].

The importance of the impact of these individual and environmental deficits on human development has aroused interest in the field of neuroscience. The contemporary neuroscientific study of poverty proposes to identify certain aspects in relation to deficits modulated by risk factors. Researchers are attempting to determine in which periods of child development risk factors have a greater impact and how these influence the execution of tasks that require cognitive processing [17]. The objective of these studies is to highlight implications in the activation of neural and behavioural networks and potential interventions to help alleviate this phenomenon [17]. The purpose of many studies has been to verify whether stimulation in different dimensions of our mental functioning, such as executive functions, translates into an improvement in the school and social environment [18,19].

There are many studies that have tried to develop strategies to intervene with children and adolescents. Interventions focused on different disciplines have been found to improve academic performance. Specifically, studies have indicated that physical exercise [20,21], use of dietary supplements [22] or social skills training have a positive impact on cognitive abilities. For example, the program by Rickel (1986) [23] focuses on promoting the use of cognitive domains, such as language, or executive functions, such as decision-making, cognitive flexibility, attention or reasoning, with the objective of improving individuals’ social cognition.

Studies that promote the improvement of cognitive skills have focused on training to achieve a general improvement [24]. The meta-analysis by Karch et al. (2013) [25] analyses cognitive training programmes in children and adolescents to improve cognitive skills such as attention or memory and executive performance, as well as behaviour or psychopathology. The effects of cognitive training were found to be small on executive function and attention, but significant on memory, behaviour and psychopathology, with the latter standing out. Regarding the training of executive functions, there are studies that seek their improvement in general [26,27] or in specific functions such as inhibitory control, memory, planning or reasoning [28,29]. The results of the programs show significant improvements in reasoning [28], cognitive flexibility, planning, metacognition and inhibitory control [26,27] and in fluid reasoning and processing [24]. The study by Giovannetti et al. [29] showed inconclusive results but highlighted the importance of considering individual and contextual differences in interventions that seek to optimize executive functions in children.

The value of cognitive training lies in its importance as an ideal element to stimulate brain plasticity. The brain maintains the ability to change throughout life, and systematic practice, such as cognitive training, is necessary for the establishment of new neural circuits and for the strengthening of synaptic connections between neurons. This capacity of the brain, known as cerebral plasticity, makes it possible to modify the morphology of neurons, which enables effective learning [14].

The objective of this systematic review is to identify and offer a synthesis of cognitive interventions in children and adolescents in Spain in which cognitive processes that have been compromised by the risk of social exclusion are trained.

## 2. Materials and Methods

### 2.1. Search Strategy

The Cochrane handbook for Systematic Reviews of Interventions [30] was used to design the protocol and the Preferred Reporting Items for Systematic Reviews and Meta-Analyses Protocols (PRISMA-P) [31] was followed to describe the protocol. The systematic review protocol was registered in the International Prospective Register of Systematic Reviews PROSPERO on 9 April 2022 with the registration number CRD42022315803: TS = ((cogniti * NEAR/3 stimulat *) OR (cogniti * NEAR/3 training) OR (cogniti * NEAR/3 intervent *) OR (cogniti * NEAR/3 enhanc *)) AND (child * OR adolescent * OR teen * OR youth * OR “school student *”) AND (income * OR socioeconom * OR econom * OR exclusion OR poverty) AND (ALL = (Spain OR españ * OR spani *)).

A first search was carried out on 8th November of 2021 in the Web of Science, and then the strategy was adapted to the following databases: PubMed, Medline, Dialnet, PsycINFO, PsycArticles, Psychology and Behavioral Sciences Collection and ERIC to obtain a large sample of interventions.

### 2.2. Article Review

After obtaining an adequate number of studies, the metadata for each were downloaded to a common Excel sheet. Duplicate results were eliminated, and the eligibility of each review was determined per the inclusion and exclusion criteria.

### 2.3. Eligibility Criteria

The following inclusion criteria were applied (see Table 1): studies written in English or Spanish in which the interventions were carried out with a Spanish population and of medium-low SES, aged 5 to 18 years, both inclusive, and interventions aimed at improving some cognitive domain or socioemotional or psychoeducational skills. Interventions delivered by psychologists, primary caregivers, teachers or qualified personnel were included. Studies with participants who showed specific performance deficiencies (for example, in mathematics or language) or behavioural problems were included. However, studies of specific interventions for any developmental disorder (for example, ADHD or autism) or physical disability were excluded. Studies that intervened only with participants of high or medium-high SES were excluded, but those who worked with a varied sample were included. There were no exclusion criteria regarding the date of the publication of the studies.

### 2.4. Selection of Studies

Once the inclusion and exclusion criteria were established, we reviewed each of the studies by title and abstract. A third author was responsible for intervening in doubtful cases. The reasons for exclusion were detailed on an Excel spreadsheet, and we finally obtained a sample of studies that would later be reviewed in full text for inclusion in the systematic review.

### 2.5. Data Extraction

Once the final sample was selected, a coding sheet was designed, taking the Patient, Intervention, Comparator and Outcome (PICO) format as a reference.

### 2.6. Data Analysis and Synthesis

Through the systematization table, the information on each of the studies was qualitatively organized, and the data were compared to analyse them together to extract the relevant aspects regarding the chosen topic.

## 3. Results

### 3.1. Identification of Studies

**Figure 1:**
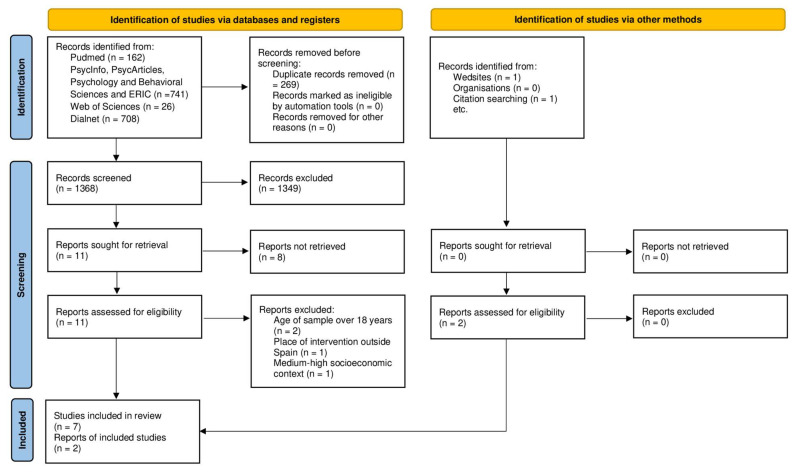
Search flowchart and selection process.

The application of the search equation in the different databases (Figure 1) produced 1637 reports published in 1992 through 2022. After eliminating the duplicate articles, 1368 articles were added, and 2 more articles were found in a manual search. After reviewing the title and abstract, a total of 1349 articles were rejected for the reasons included in Table 1. Then, the full text of the remaining 11 articles was read (Figure 1).

### 3.2. Characteristics of the Studies

The information related to the selected studies is presented in Table 2 and Table 3.

#### 3.2.1. Population

Sample size varied from studies with a low number of participants (e.g., *n* = 12; [32]) to studies with larger sample sizes (e.g., *n* = 283; [33]).

In the interventions chosen for the review, the age range of the sample varied between 5 and 20 years. A study with an age range of up to 20 years was chosen because the sample also included minors and it was not possible to separate participants from each other. Two types of populations were observed: one was childhood and early adolescence, with ages ranging from 5 to 12 [18,19,33,34,35,36], and one was adolescence, with ages ranging from 13 to 20 [32,37].

The socioeconomic level of the study sample was low or medium-low. The study by Pozuelos-López [36] considered a varied sample in which a high socioeconomic level was also included. Only in one case, in the study by de la Morena [34], was the family context risky, and the participants were in social protection centres.

Regarding the sample conditions, only the studies by Mata et al. [35], Pozuelos-López [36] and Sánchez-Pérez et al. [19] excluded participants with a history of chronic diseases, special needs or psychopathological diagnosis. 

#### 3.2.2. Interventions

Although all the included studies had the main objective of improving the cognitive performance of the participants, two types of subobjectives were differentiated to evaluate the effectiveness of this intervention through the improvements observed in the minors and to evaluate the improvement of their cognitive abilities.

Regarding the type of interventions in the literature, there were cognitive training studies, computerized on two occasions with self-instruction or metacognitive training on another two and with gamification in another, and psychoeducational interventions.

Through these trainings, the improvement of some cognitive dimensions was sought, such as social cognition, language, executive functions, decision-making, flexibility, working memory, inhibitory control, branching or multitasking, attention and planning or reasoning. In addition, the studies by Tellado [38] and Mata et al. [35] examined improvements in mathematical skills. The psychoeducational interventions in these studies were aimed at training and teaching the participants in a compensatory way, since they sought to solve problems derived from learning. The interventions provided the youth with resources to develop their skills in a way that allowed them to acquire knowledge autonomously; the interventions ensured the well-being of the individuals in the performance of day-to-day tasks.

Regarding the format of the intervention, most studies applied interventions to subjects in groups (six studies). In the study by Sanz de Acedo and Iriarte [37], the group and individual format were combined; in the study by Pozuelos-López [36], the format was individual; and in the Sáiz-Manzanares and Román-Sánchez [18] study, this information is not available.

Regarding the intervening agent, the studies are divided into external or internal agents. In cases where the agent was external (such as a researcher or psychologist), a professor from the centre provided complementary help in the training or evaluation of the participants. Regarding the rest, this information is not available. Seven of the studies intervened in the educational centre during school hours and only in one in the afternoon after school. The aim was to eliminate certain variables, such as those derived from a work environment different from the usual for participants.

Regarding the duration of the intervention, the shortest was that of Pozuelos-López [36], and the longest was that of Sanz de Acedo and Iriarte [37], with 3 weeks and 9 months of duration, respectively.

#### 3.2.3. Comparison

Regarding the design of the studies, quasi-experimental [33,35,37,38], longitudinal [19,34] and nonequivalent control group designs [18] were used. In two of the studies, this information was not available [32,36].

Regarding the timing of evaluations in the studies, all evaluated pretraining and posttraining dependent variables, but in some studies, an evaluation [19,38] and/or follow-up was added [18,34,38].

#### 3.2.4. Outcomes

In general, significant improvements are observed in trained cognitive functions, as the ones listed in the following subsections.

##### Decision-Making

In the studies of Mata et al. [35], Sanz de Acedo and Iriarte [37], and Sáiz-Manzanares and Román-Sánchez [18], in which decision-making was studied, the trained group demonstrated significant improvements compared with the untrained group with values of *p* = 0.0001; *p* < 0.001; and *p* = 0.0001, respectively. In the last study, these positive results are found in the follow-up measures.

##### Planning

Regarding executive function, [18] found significant differences in the planning measures of the training group with respect to the control group (*p* = 0.0253) in the follow-up measures. In the study by Vita-Barrull et al. [33], they found a significant reduction in planning dysfunction compared with the time prior to the intervention (*p* < *0*.001). In this case, Tellado [38] trains this function in two groups obtaining good results for both, but significant improvement occurs in the group with the worst initial results (difference of 20.83%), and better results were observed over time.

##### Cognitive Flexibility

The studies by Sáiz-Manzanares and Román-Sánchez [18] and Sanz de Acedo and Iriarte [37] show a significant improvement in the scores of the training group in cognitive flexibility when compared with the control group, with values of *p* = 0.0114 and *p* < 0.001, respectively. In the Sáiz-Manzanares and Román-Sánchez [18] study, these results can be observed in the follow-up measures, whereas in the Sanz de Acedo and Iriarte [37] study, they can be seen during the posttraining. In the study by Vita-Barrull et al. [33], they found a significant reduction in cognitive flexibility dysfunction compared with the time prior to the intervention (*p* < 0.001).

##### Working Memory

In their study, Sánchez-Pérez et al. [19] did not obtain significant results in the evaluation measures of this process. However, working on this function helped improve the results of the participants’ mathematics learning. That is, a transfer effect was achieved. In contrast, in the study by Pozuelos-López [36], significant improvements in working memory were obtained after training (*p* < 0.05). Another study that also achieved good results in working memory (*p* < 0.001) with respect to the pre-intervention time was that of Vita-Barrull et al. [33].

##### Inhibition

In the studies of Sánchez-Pérez et al. [19], Sanz de Acedo and Iriarte [37], Vita-Barrull et al. [33] and Pozuelos-López [36], significant improvements were observed in participants who trained the executive function of inhibition. In the study by Sánchez-Pérez et al. [19], improvements were perceived in inhibition tasks 1 (*p* = 0.030), 2 (*p* = 0.004), 3 (*p* = 0.002) and Nogo (*p* = 0.001). Sanz de Acedo and Iriarte [37] and Vita-Barrull et al. [33] obtained an effect size of *p* < 0.001 in their study and Pozuelos-López [36] obtained an effect size of *p* < 0.01 in the posttraining measures.

##### Multitasking

In the study by Sánchez-Pérez et al. [19], this process is considered, but no significant results are obtained.

##### Language

There are two studies that work on language among other cognitive functions. In the case of the study by Gil-Calvo [32], oral and written expressions are studied, and satisfactory results are obtained. However, the measure used in this study is observational, so these results should be interpreted with caution. Sánchez-Pérez et al. [19] obtained satisfactory results (*p* = 0.002), so we observed the effectiveness in the posttraining.

##### Reasoning and Perception

In the study by Gil-Calvo [32], there is an improvement in the reasoning process and in the perception process among participants. However, the measures used in this study are observational, so these results should be interpreted with caution.

##### Social Cognition

In the studies reviewed, social cognition, as well as interpersonal cognition, are approached in a psychoeducational way. In the studies of Mata et al. [35], with *p* = 0.037, Sáiz-Manzanares and Román-Sánchez [18], with *p* = 0.0002, and de la Morena [34], satisfactory results in the measurements of this variable were obtained; although in the case of the latter, no comparative data are provided for the two groups in posttraining. In the study by Vita-Barrull et al. [33], they work like the rest of the functions and work from emotional control, obtaining good results (*p* < 0.001).

##### Learning in Mathematics, Reading and Writing

Mathematics, in the studies of Sánchez-Pérez et al. [19], Tellado [38], and Pozuelos-López [36], and language, in the study by Sánchez-Pérez et al. [19], have been skills improved through transfer effects by cognitive function training. The training of these functions, such as attention [36], working memory [19] and planning [38], implemented through self-instruction, led to an improvement in mathematical skills in the case studies by Tellado [38] and Pozuelos-López [36] and an improvement in reading in the case study by Sánchez-Pérez et al. [19].

## 4. Discussion

This systematic review aimed to synthesize the scientific literature on interventions in cognitive domains for children and adolescents living in disadvantaged contexts in Spain. Nine intervention studies were included in which different cognitive dimensions were addressed and from which ways to improve the cognitive performance of children might be extracted.

- Although there is a scarcity of studies carried out in Spain on the subject, interesting results have been obtained in this review.
- The interventions analysed show, in general, an improvement in the development of cognitive skills in children with low SES. However, due to the heterogeneity between some interventions and others, this statement should be interpreted with caution. It is essential to study in more detail which variables could influence the results obtained.

It has been observed that, in general, the hypotheses of the studies tried to show a significant difference between a control group, with or without low SES, and a training group with low SES. The results obtained have fully or partially confirmed these hypotheses. However, certain characteristics should be considered:

- In addition to a low SES, some studies also include a population with vulnerable conditions, such as behavioural problems or learning difficulties [32,37,38]. It is relevant that these interventions were successful since it is an important step for the population with these difficulties; often, we do not focus on their strengths and potentialities. These data are consistent with international scientific evidence. For example, Peijnenborgh et al. [39], in their systematic review of cognitive training in children and adolescents with learning difficulties, show short-term improvements in verbal working memory, visuospatial working memory and decoding of words as results of this training. Similarly, in the study by Rueda et al. (2021) [40], they state that training of cognitive skills such as working memory, executive attention and cognitive flexibility produces improvements in performance on these tasks, and these benefits appear to be greater for children with lower initial levels of cognitive skills.
- In relation to this, the study by Tellado [38] compared a sample of children with low levels of planning with a sample with high levels of planning after training, indicating that “those who needed it most gained more” (p. 249). This statement shows the importance of including children with difficulties in the interventions, since they obtain even greater benefits than the rest of the population. Interventions such as those of Sanz de Acedo and Iriarte [37] and Gil Calvo [32] also show significant improvements among vulnerable populations after training.

The effect of the interventions can also be compromised depending on, first, the age of the sample, and second, the skills trained. These variables can be modulated in the improvement of cognitive abilities due to cognitive plasticity, which varies depending on the life cycle [41] and the existence of sensitive periods in relation to moments especially important for early stimulation [12]. Thus, there are differences in the efficacy of the interventions as a function of the age of the sample. For example, Sanz de Acedo and Iriarte [37] indicate that the age of their sample helped in achieving their objectives, since the cognitive nature of the tasks trained required a higher level of assimilation, difficult to achieve for younger individuals. In contrast, Mata et al. [35] emphasize the importance of differentiating the interventions by age, since the existence of disparate age ranges in their sample could be a possible variable influencing the significance of the data.

Regarding these aspects, the analysis of the review by Peijnenborgh et al. [39] showed a greater benefit in children older than 10 years in verbal working memory. This evidence is also supported by Rosselli et al. [42], who state that the development of executive functions, such as inhibitory control, planning or cognitive flexibility, is gradually better as the child gets older and reaches an adult level around the age of 10 years. According to the literature, cognitive functions can be seen in children at approximately 3 years of age, performance improves through age 5 and reaches an adult level at age 10 in general [42]. Therefore, considering the age range of the samples of the studies in this review, ages 5 through 13, and trained skills, such as planning, working memory, inhibition and flexibility, there is consistency in the results, since the prefrontal cortex of children achieves greater development from 7 to 12 years of age and, therefore, better performance in these cognitive functions [43]. Thus, if the interventions are adequately adapted to the age of the participants, they can be fruitful for both children and adolescents.

In addition to executive functions, some of the interventions addressed the social cognition of minors, obtaining positive results [18,33,34,35,36]. Authors such as Slavin and Lake [44] emphasize the importance of the inclusion of work in social cognition, as well as in metacognitive strategies in interventions with children, since the effect sizes reported by this type of training are greater than in the rest of the programs [44]. In the case of minors with low SES, it seems evident to influence the work of social cognition, since it refers to the processing of relevant information to carry out social interactions [45]. Given that in the environments in which these minors operate, on many occasions, an insufficiently structured context is perceived that allows a regulated learning of social codes due to social exclusion and the lack of environmental stimulation, training in this dimension can be very effective.

In the same way, language training is essential in the case of minors belonging to families with low SES, as seen in the studies of Sánchez-Pérez [19] and Gil-Calvo [32]. It was evident in their studies that low SES had negative effects on their subjects’ speech and writing.

Among the studies, a difference is observed in terms of the agents involved in the intervention process. For example, in the studies of Sánchez-Pérez et al. [19], Sáiz-Manzanares and Román-Sánchez [18], and Gil-Calvo [32], an internal agent (e.g., teacher in the centre) led the intervention. This entails positive and negative aspects, but ideally, it would be an exercise that would consider both internal and external professionals. The joint work of both would facilitate the implementation of the programs and would achieve, in the long term, greater generalization. Therefore, implementing programs of this type in which professionals are needed in schools as a new methodology would imply the need to design new educational policies [19].

The participation of cotherapists, such as fathers, mothers or relatives, could help develop better results, since the training would affect not only the school context of the children but also the family context. Thus, the implementation of these programs during school hours and together with the help of family members would enable better development of the child, since interventions would not be conducted in environments more typical of a laboratory but in surroundings familiar to the children. This understanding between professionals, teachers and family members would add greater ecological validity to the data, since the improvements perceived in the training would be generalized and shown in all the systems that involve the development of the child.

Finally, only three studies present a follow-up measurement for their dependent variables [18,34,38], and the results of these three studies are very positive. The findings indicate that, in addition to good results after training, improvements are maintained for longer periods of time. This, in addition to assuming a very clear benefit for the participants, is encouraging for the implementation of cognitive intervention programs in the school curriculum.

### Limitations and Future Directions

This systematic review has limitations related to the applied methodology.

First, access to the metadata in some databases was difficult, so the preparation of the Excel spreadsheet was manual, lending itself to human error. In addition, access to the full text of some studies was difficult. In the case of doctoral theses, these could not be obtained due to a lack of time and resources.

Second, due to the characteristics of some studies, it was not possible to dissociate sample subgroups in studies that included an older sample, a criterion that is not within the objective of this review.

After this synthesis of results, future lines of research are proposed that may help research professionals improve the cognitive processes of at-risk minors.

In general, it has been shown that cognitive training, in social skills and physical exercise, contributes to improvements in the development of cognitive functions of children with low SES. However, little is known about how diet could favour the development of these functions, since the only study on the subject did not show significant results [22]. Due to the importance of a good diet in the development of minors and considering that living with a low SES brings with it deficiencies in access to basic resources, such as food, it is understood that interventions should start here. The importance of meeting the physiological needs of people is paramount, since it is difficult for a person to worry about improving cognitive functions when his or her basic physiological needs are not covered [46]. Therefore, we recommend investigations into the implementation of nutritional intervention programs to improve the quality of life of children and, therefore, their cognitive development.

It has also been observed that there are evolutionary periods that are especially conducive to the development of each cognitive function and that this development depends on many other variables in addition to age. However, in the studies analysed, interventions were not performed to study this development in each age range. This suggests the need to conduct studies in which the sample is divided by more limited evolutionary periods and the cognitive dimensions are differentiated. Defining the interventions by evolutionary periods will help determine which interventions are most effective for each child and in which evolutionary period a better result will be achieved.

There is a need to conduct more intervention studies in Spain, given the small number of interventions that were found.

## 5. Conclusions

This review has shown the heterogeneity that exists in terms of training aimed at improving cognitive dimensions for children and adolescents. From this systematic review, the following findings can be highlighted:

- Studies focused on the improvement of cognitive functions in children is very scarce in Spain, but the differences in the execution of the different studies found, can provide us with knowledge about different variables that influence the effectiveness of these interventions, specifically:
- Children with low family SES obtain greater benefits from this type of intervention, since these children manage to develop their cognitive functions significantly compared with a population with a high or medium SES.
- Performing an intervention that considers the different age ranges among minors is essential to developing better training and achieving better results.
- There is a great variety of cognitive functions that have been trained. Thus, in the development of these training programs, the importance of choosing those most appropriate based on the age and conditions of vulnerability of the sample is emphasized.
- Variables such as intervention agents or cotherapists involved in the development of interventions may influence the training results of children and adolescents.

## Figures and Tables

**Table 1:** Inclusion and exclusion criteria.

Criterion	Inclusion Criteria	Exclusion Criteria
Population	Studies that include children and adolescents aged 5 to 18, both inclusive	The rest of the population
Intervention	Cognitive, socioemotional, social or communication skills domains, physical exercise or nutritional supplements	Specific for developmental disorders, physical or mental disabilities
Design	All types of studies	Not applicable
Publication type	Academic publications, doctoral theses	Other types of educational or informative publications
Intervention context	All kinds of contexts	Not applicable
Cultural context	At least one group of participants with medium-low or low SES	Only participants of high, medium-high SES

**Table 2:** Systematization of the studies: General information.

Study	Population				Intervention
Authors	Sample	Groups	Age Range	Added Adversities	Intervention Objective
Mata et al., 2018	N = 61	Three groups: group with low SES * attending the program (*n* = 18); group with low SES not attending the program (*n* = 16) and group with medium-high SES attending the program (*n* = 27)	7–12	NS **	Evaluate the effectiveness of a training program
Sánchez-Pérez et al., 2018	N = 104	Two groups: training group with low SES (*n* = 51) and control group with medium-high SES (*n* = 53)	7–12	NS	Improve cognitive skills
Sáiz- Manzanares and Román Sánchez, 1996	N = 25	Two groups: training group with low SES (*n* = 13) and control group with low SES (*n* = 12)	5–7	NS	Improve cognitive skills
Sanz de Acedo and Iriarte, 2001	N = 109	Two groups: training group with medium-low SES (*n* = 50) and control group with medium-low SES (*n* = 59)	14–15	Behavioural problems, learning difficulties and little motivation to study	Evaluate the effectiveness of a training program
Tellado, 2001	N = 25	Two groups: training group with low level of planning and medium-low SES (*n* = 12) and training group with high level of planning and medium-low SES (*n* = 13).	9–13	Mathematical difficulties or delays in the area of mathematics	Improve cognitive skills
Pozuelos-López, 2014	N = 97	Two groups: group with metacognitive feedback with medium-low SES (*n* = NS) and performance feedback group with medium-low SES (*n* = NS).	NS	NS	Improve cognitive skills
De la Morena-Fernández, 2016	N = 24	Two groups: training group with low SES (*n* = 12) and control group with low SES (*n* = 12)	5–10	Economic difficulties and social and family isolation	Evaluate the effectiveness of a training program
Gil-Calvo, 2010	N = 12	A group: participants with low SES	16–20	Special educational needs, little knowledge of the Spanish language, unstructured behaviours and behavioural and conceptual deficiencies	Improve cognitive skills
Vita-Barrull, et al., 2021		Four groups: two gamified groups with low SES (*n* = 176) and two non-gamified groups with low SES (*n* = 107)	6–13	NS	Evaluate the effectiveness of a training program

* SES: Socioeconomic level; ** NS: Not specified.

**Table 3:** Systematization of the studies: Specific information.

Study	Intervention			Comparison	Results
Authors	Type of Intervention	Cognitive Functions Worked	Intervention Agent and Intervention Schedule	Impact Assessment	Results
Mata et al., 2018	Cognitive training and psychoeducational intervention	Executive functions (decision-making) and social cognition	External agent (psychologist); Afternoons	Pretraining and posttraining evaluation	In comparison with the group of children with low SES * who did not attend the program, the children with low SES who attended the program showed significant increases in the measures of the dependent variable: decision-making (*p* = 0.0001). In comparison with the group of children with medium-high SES attending the program, children with low SES attending the program showed significant increases in the measures of the dependent variable: social cognition (*p* = 0.037).
Sánchez-Pérez et al., 2018	Computerized cognitive training	Executive functions (working memory, inhibition and multitasking), learning (mathematics and reading)	Internal agent (faculty); School hours	Pretraining, training and posttraining evaluation	In comparison with the control group with medium-high SES, the training group with low SES shows significant increases in the measures of the dependent variables: mathematics (*p* = 0.000); language (*p* = 0.002); inhibition type 1 (*p* = 0.030), type 2 (*p* = 0.004), type 3 (*p* = 0.002) and Nogo (*p* = 0.001)
Sáiz-Manzanares and Román Sánchez, 1996	Cognitive training	Executive functions (planning, decision-making and flexibility) and interpersonal cognition	Internal agent (faculty); School hours	Pretraining, posttraining and follow-up evaluation	In comparison with the control group, the training group shows significant increases in the measures of the dependent variables: flexibility (*p* = 0.0114); decision-making (*p* = 0.0001); social cognition (*p* = 0.0002) and planning (*p* = 0.0253)
Sanz de Acedo and Iriarte, 2001	Cognitive training	Executive functions (decision-making, inhibition and cognitive flexibility)	External agent (researcher); School hours	Pretraining, training, post	In comparison with the control group, the training group shows significant increases in the measures of the dependent variables: flexibility (*p* < 0.001), decision-making (*p* < 0.001) and inhibition (*p* < 0.001).
Tellado, 2001	Cognitive training	Executive functions (planning)	External agent (researcher); School hours	Pretraining, training, posttraining and follow-up evaluation	Significant increases in planning scores are shown in both groups (*p* < 0.00). However, the difference in the total percentage of change between the group of subjects with low planning and those of the group with high planning is 20.83%.
Pozuelos-López, 2014	Computerized cognitive training	Executive functions (inhibition and working memory)	NS agent; School hours	Pretraining and posttraining evaluation	In comparison with the performance feedback group, the group with metacognitive feedback shows significant increases in the measures of the dependent variables: inhibition (*p* < 0.01) and working memory (*p* < 0.05).
De la Morena-Fernández, 2016	Psychoeducational intervention	Social cognition	External agent (researcher); School hours	Preintervention evaluation and postintervention follow-up	In comparison with the control group, the intervention group shows significant increases in the measures of the dependent variable: social cognition.
Gil-Calvo, 2010	Cognitive training and psychoeducational intervention	Executive functions: reasoning; Language: oral and written expression; Perception	Internal agent; School hours	NS **	NS
Vita-Barrull, et al., 2021	Cognitive training	Executive functions (planning, flexibility, working memory inhibition) and social cognition (emotional control)	Internal agent (faculty); School hours	Pretraining and posttraining evaluation	In comparison with the gamified groups, the non-gamified groups show significant decreases in the measures of the dependent variables: planning (*p* < 0.001), flexibility (*p* < 0.001), working memory (*p <* 0.001), inhibition (*p* < 0.001) and emotional control (*p* < 0.001)

* SES: Socioeconomic level; ** NS: Not specified.
